# Supplementary material for: Projected loss of brown macroalgae and seagrasses with global environmental change
Source: Nat Commun. 2024 Jun 24;15:5344. doi: 10.1038/s41467-024-48273-6 (PMC11196678; doi:10.1038/s41467-024-48273-6)
Supplement: Supplementary file 1 — Supplementary Information [file 41467_2024_48273_MOESM1_ESM.pdf]

Projected loss of brown macroalgae and seagrasses with global environmental change

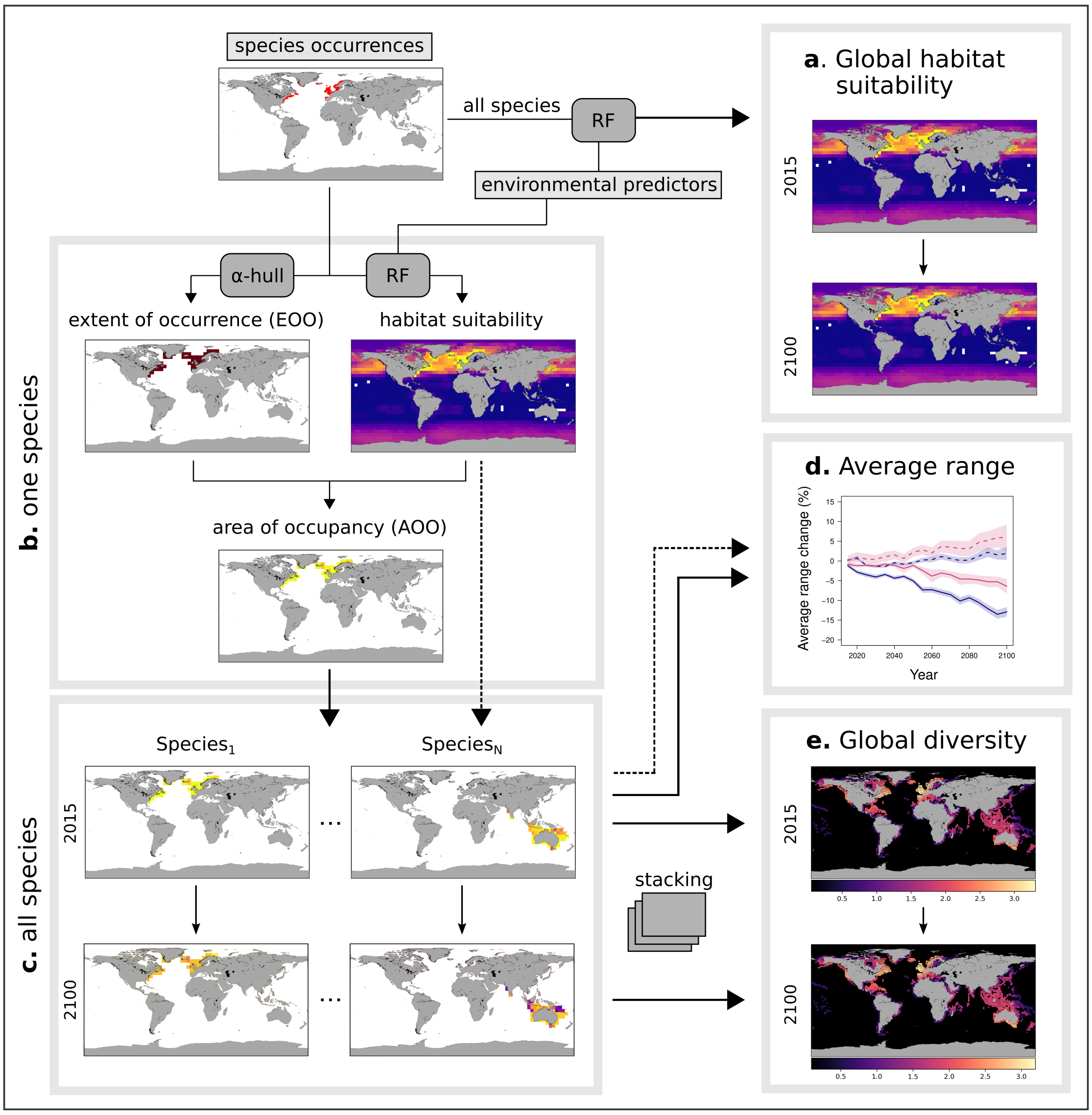

Supplementary Fig. 1 Analytical workflow. We combined species occurrence records with environmental and climatic layers (temperature, salinity, primary productivity, light, ice-cover, depth) to generate global habitat suitability maps (**a**) and species-specific habitat suitability maps (**b**) using a random forest algorithm. We filtered both to exclude cells with limiting light conditions for macrophyte photosynthesis (see Methods, not shown). We clipped species-specific habitat suitability maps with the *extent of occurrence*[1] generated from species occurrences with an *α*-hull procedure to obtain the *area of occupancy*[1] of each species (**b**). We computed areas of occupancy for all 207 macrophyte species from 2015 to 2100 (**c**), which we used to calculate average

macrophyte area-of-occupancy ("range" in panel **d**) trajectories (**d**), and also stacked to generate global maps of macrophyte species diversity (**e**). We used individual habitat suitability maps (dashed lines) to compute average macrophyte range trajectories under a "proportional-expansion" hypothesis (**d**, see Methods). We computed results in a, d, and e separately for brown macroalgae (185 species) and seagrasses (22 species).

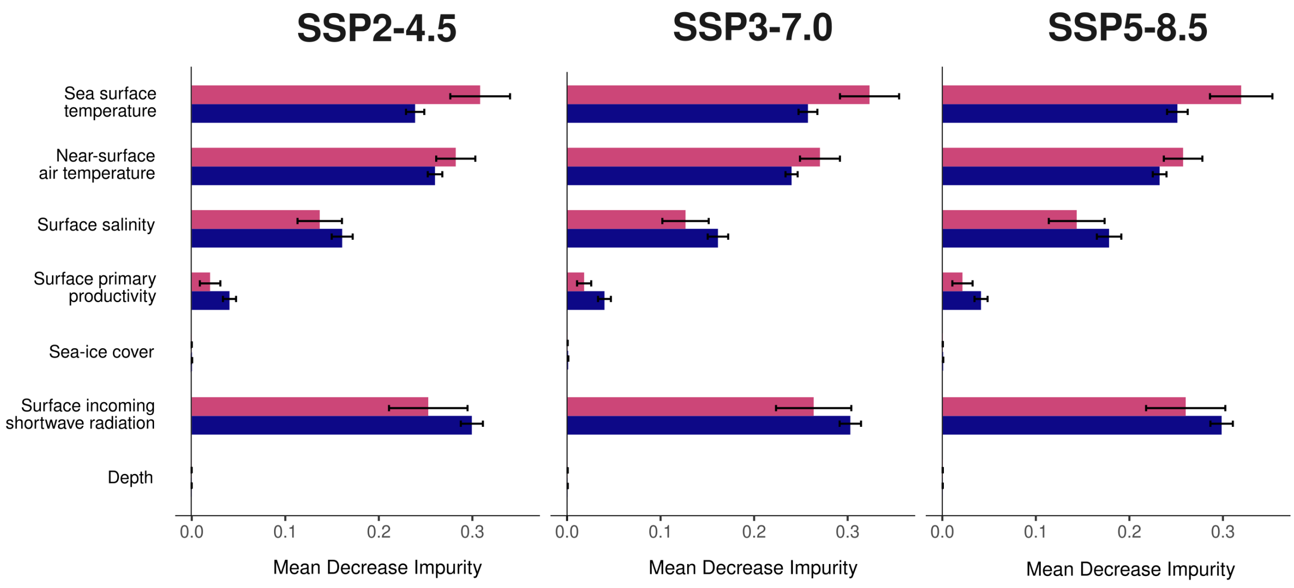

Supplementary Fig. 2 Variable importance for the species-specific random forest models. We computed variable importance as mean decrease impurity for all 207 species-specific models and then averaged among all 185 brown macroalgal and 22 seagrass species, respectively, for each emissions scenario (SSP2-4.5, SSP3-7.0, SSP5-8.5). Coloured bars represent the mean decrease impurity for brown macroalgae (purple) and seagrasses (pink). Error bars show the standard error.

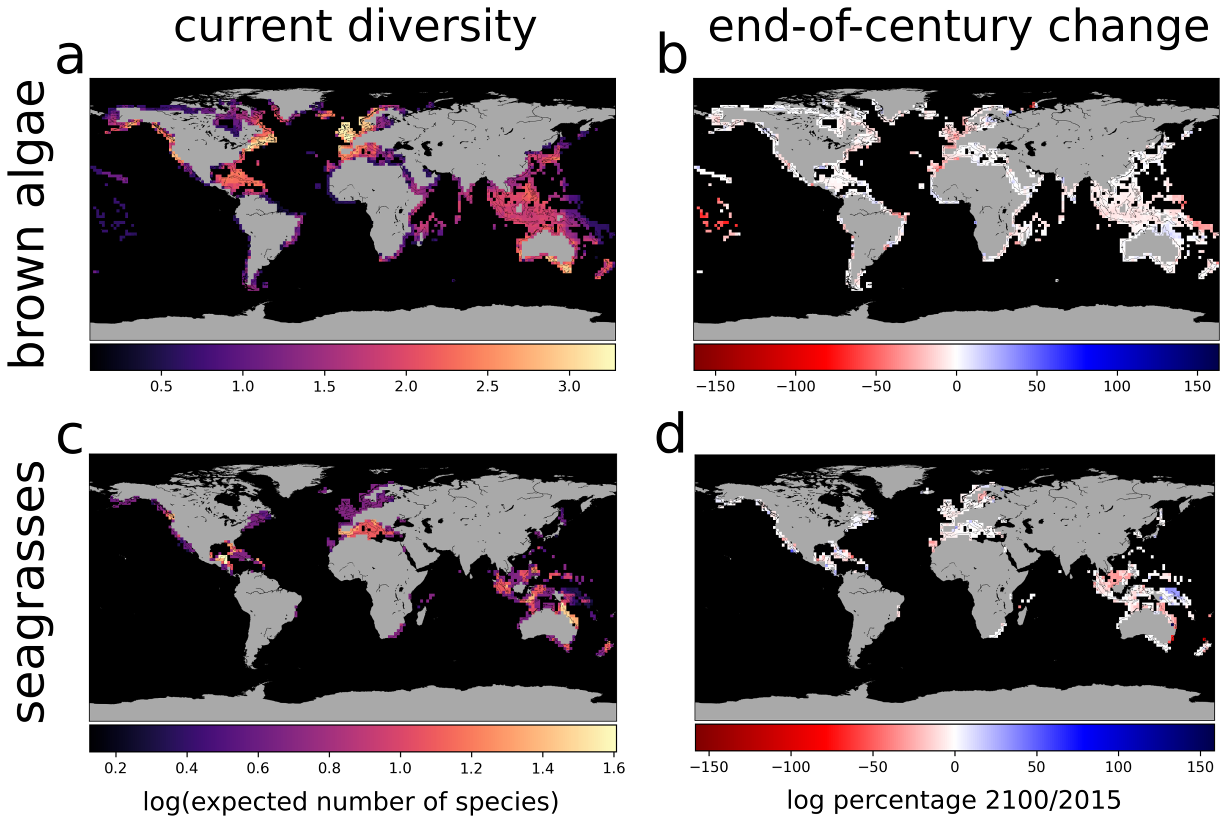

Supplementary Fig. 3 Current distribution and projected end-of-century changes in global macrophyte species diversity (emissions scenario SSP2-4.5). Panels **a**,**b** show brown macroalgae, panels **c**,**d** show seagrasses. We obtained present-day diversity (**a**,**c**) by stacking individual area-of-occupancy maps (see Methods). We computed end-of-century change (**b**,**d**) relative to 2015 using the $\log_e$ percentage[2] change ($100 \times \log_e[\text{diversity}_{2100}/\text{diversity}_{2015}]$) to show diversity losses and gains on a symmetrical scale. Gains in species diversity are shown in blue, losses in red. Species distribution data to generate the maps include 185 species of brown macroalgae and 22 seagrass species. Maps upscaled to a 2° × 2° latitude/longitude resolution to ease visualization. All maps were generated with the package *Basemap* in Python 3.

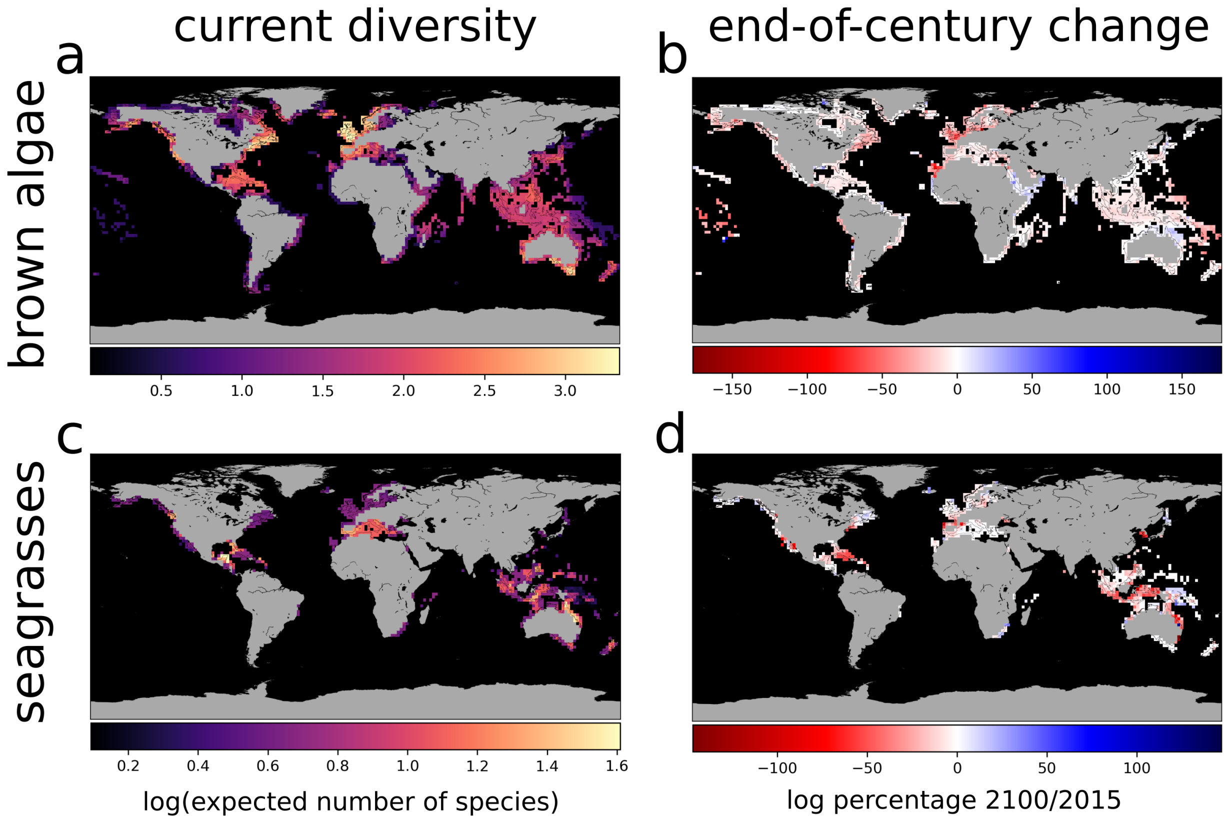

Supplementary Fig. 4 Current distribution and projected end-of-century changes in global macrophyte species diversity (emissions scenario SSP5-8.5). Panels **a**,**b** show brown macroalgae, panels **c**,**d** show seagrasses. We obtained present-day diversity (**a**,**c**) by stacking individual area-of-occupancy maps (see Methods). We computed end-of-century change (**b**,**d**) relative to 2015 using the $\log_e$ percentage[2] change ($100\times\log_e[\text{diversity}_{2100}/\text{diversity}_{2015}]$) to show diversity losses and gains on a symmetrical scale. Gains in species diversity shown in blue, losses in red. Species distribution data to generate the maps include 185 species of brown macroalgae and 22 seagrass species. Maps upscaled to a 2° × 2° latitude/longitude resolution to ease visualization. All maps were generated with the package *Basemap* in Python 3.

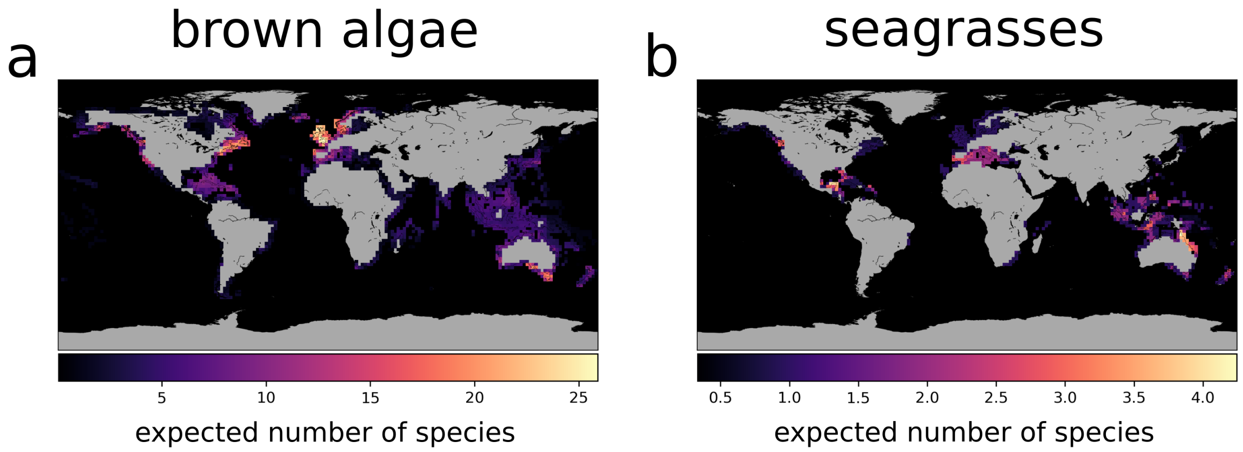

Supplementary Fig. 5 Current global macrophyte species diversity. We obtained present-day diversity by stacking individual area-of-occupancy maps (see Methods). **a** brown macroalgae, **b** seagrasses. Unlike Fig. 1, the expected number of species shown in the figure is on a linear scale. All maps were generated with the package *Basemap* in Python 3.

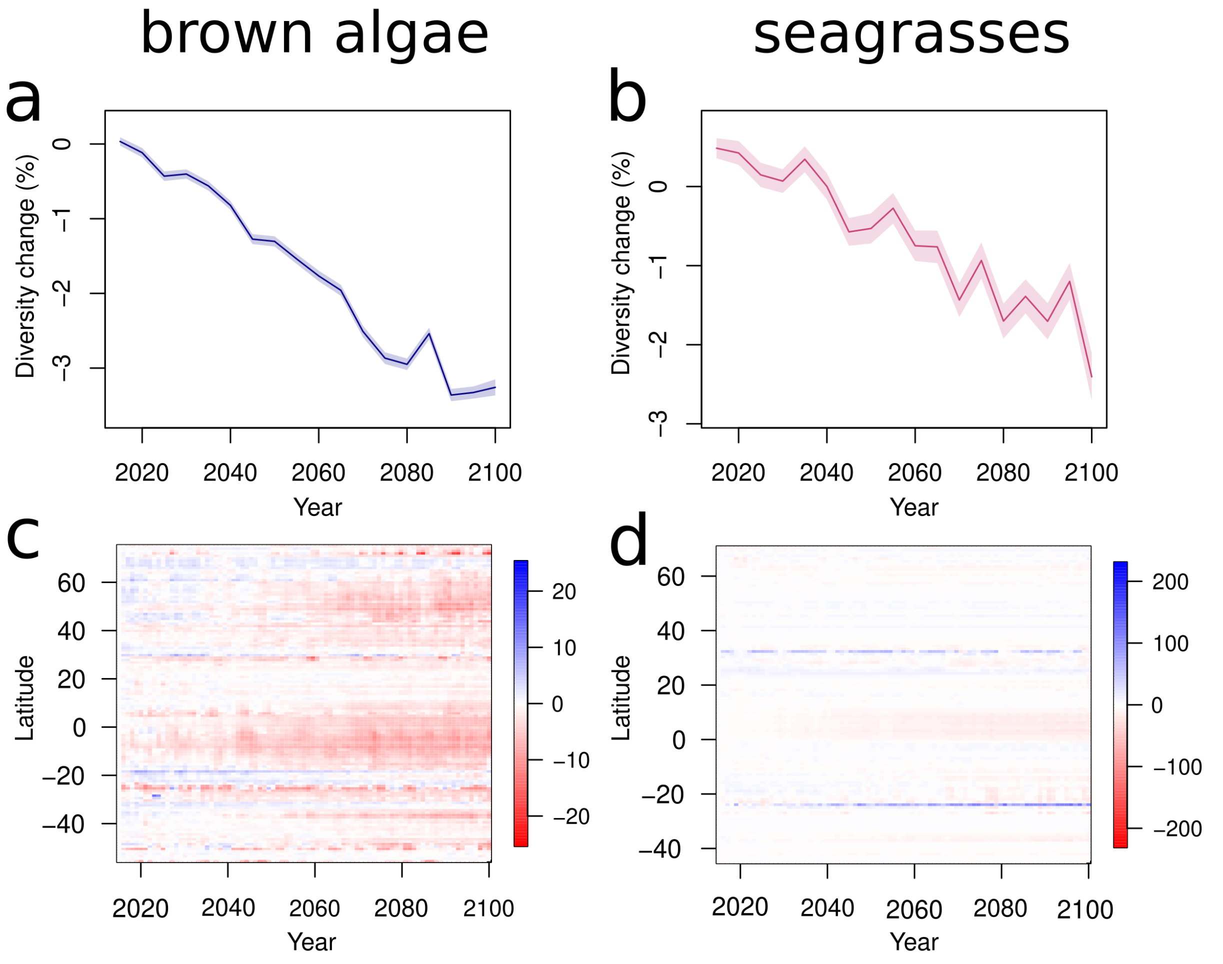

Supplementary Fig. 6 Trajectories and latitudinal trends of future changes in macrophyte diversity (SSP2-4.5). Panels **a**,**c** refer to brown macroalgae, and panels **b**,**d** refer to seagrasses. We computed change as $\log_e$ percentage change[2] in the 2100 diversity compared to 2015 diversity ($100\times\log_e[\text{diversity}_{2100}/\text{diversity}_{2015}]$). Upper panels (**a**,**b**) report the mean trajectories (solid lines) and the 95% confidence interval (shaded area) in local macrophyte diversity (i.e., number of macrophyte species in every 0.5° × 0.5° latitude/longitude grid cell) relative to 2015. We aggregated data at 5-year intervals. Lower panels (**c**,**d**) show expected future changes in diversity as $\log_e$ percentage change relative to 2015 diversity averaged across latitudes (0.5° × 0.5° latitude/longitude resolution). Gains in species diversity shown in blue, losses in red.

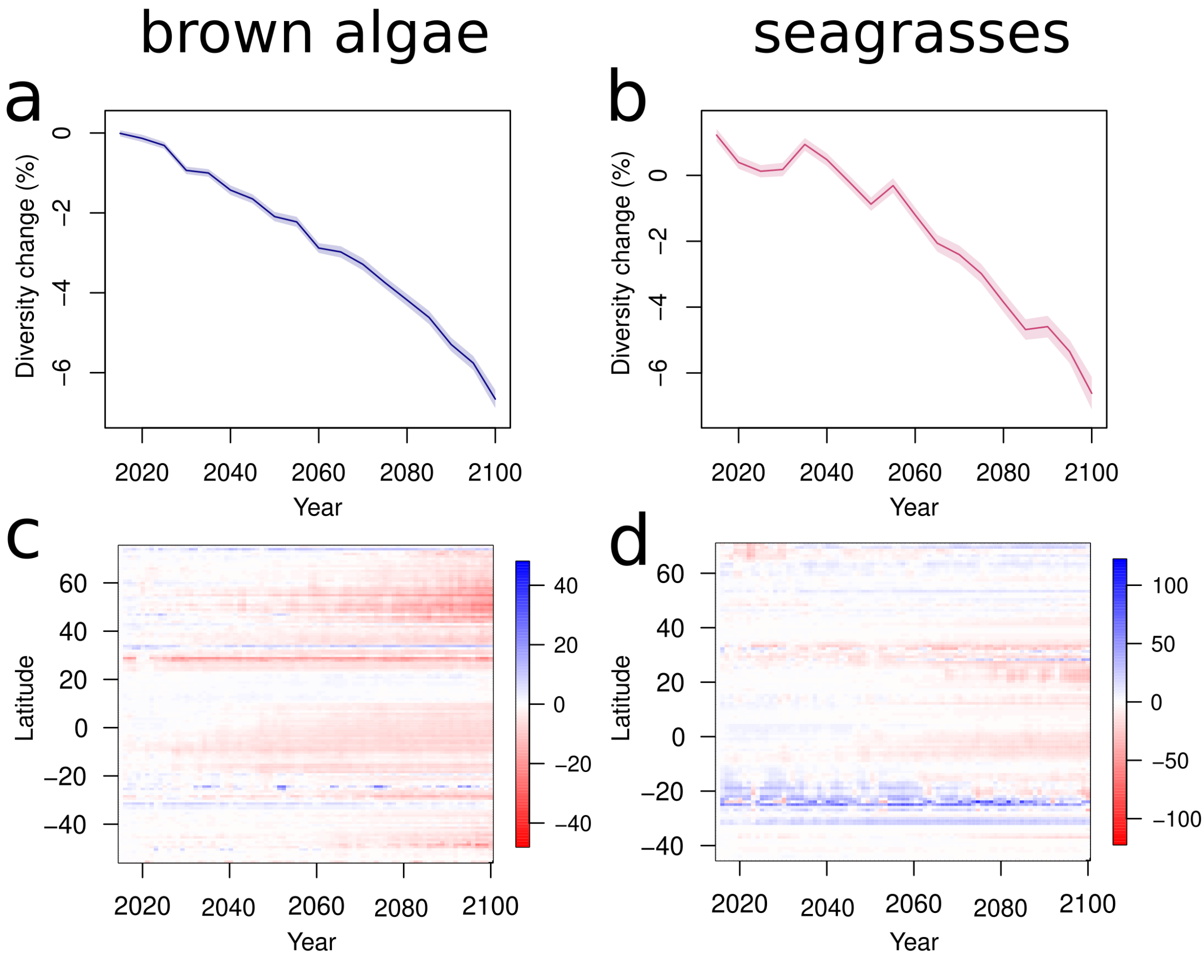

Supplementary Fig. 7 Trajectories and latitudinal trends of future changes in macrophyte diversity (emissions scenario SSP5-8.5). Panels **a**,**c** refer to brown macroalgae, and panels **b**,**d** refer to seagrasses. We computed change as $\log_e$ percentage change[2] in the 2100 diversity compared to 2015 diversity ($100\times\log_e[\text{diversity}_{2100}/\text{diversity}_{2015}]$). Upper panels (**a**,**b**) report the mean trajectories (solid lines) and the 95% confidence interval (shaded area) in local macrophyte diversity (i.e., number of macrophyte species in every 0.5° × 0.5° latitude/longitude grid cell) relative to 2015. We aggregated data at 5-year intervals. Lower panels (**c**,**d**) show expected future changes in diversity as $\log_e$ percentage change relative to 2015 diversity averaged across latitudes (0.5° × 0.5° latitude/longitude resolution). Gains in species diversity shown in blue, losses in red.

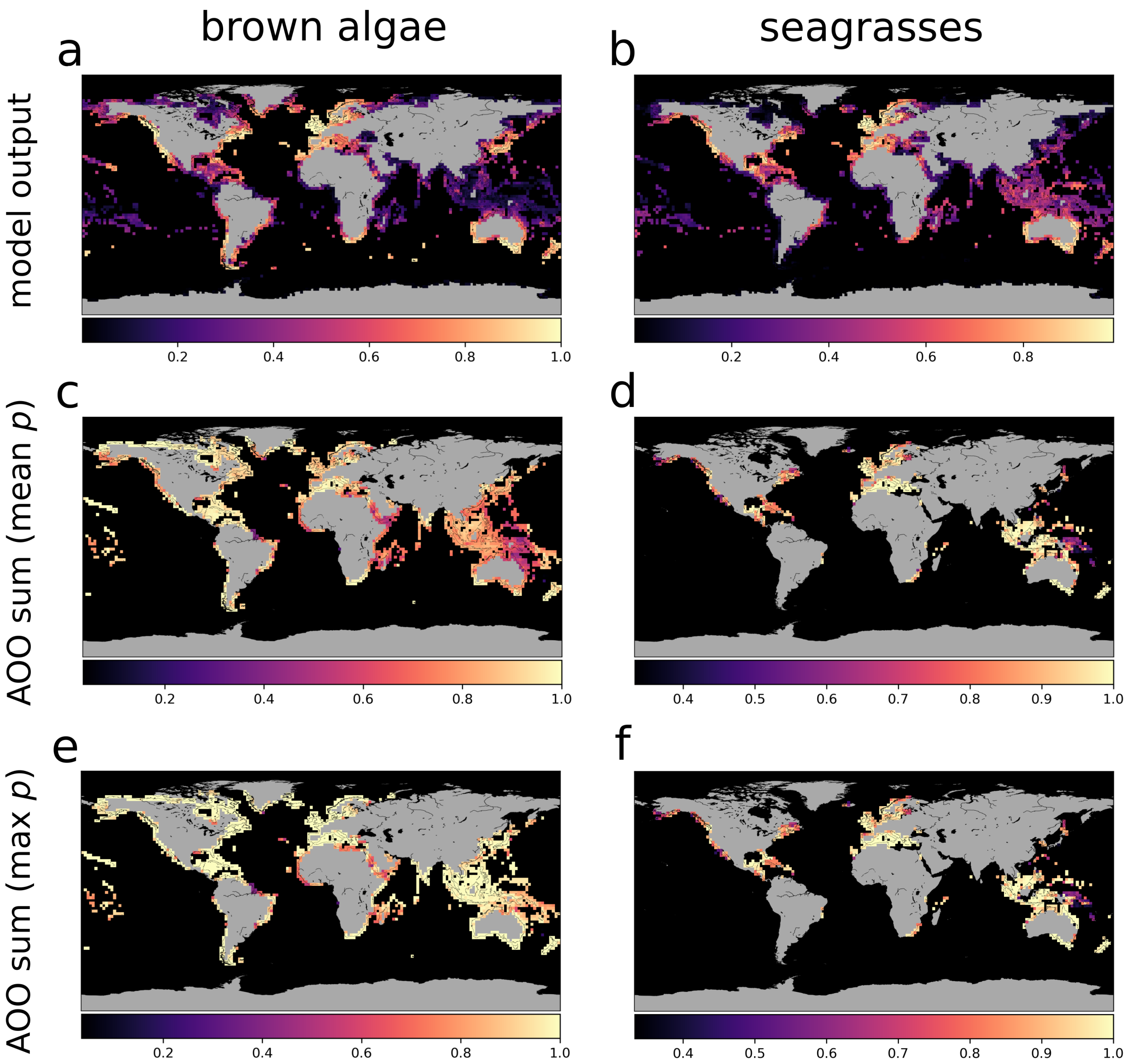

Supplementary Fig. 8 Global macrophyte habitat suitability maps obtained with different approaches (year 2015, scenario SSP3-7.0). a–b maps obtained from the global habitat suitability model we used; **c–d** maps obtained from the sum of species-specific area-of-occupancy (AOO) maps, considering the mean suitability value (p) per cell; **e–f** maps obtained from the sum of species-specific area-of-occupancy maps, considering the maximum suitability value (p) per cell. **a**,**c**,**e** brown macroalgae (n = 185 species); **b**,**d**,**f** seagrasses (n = 22 species). Coloured scalebars show relative habitat suitability (p). All maps were generated with the package *Basemap* in Python 3.

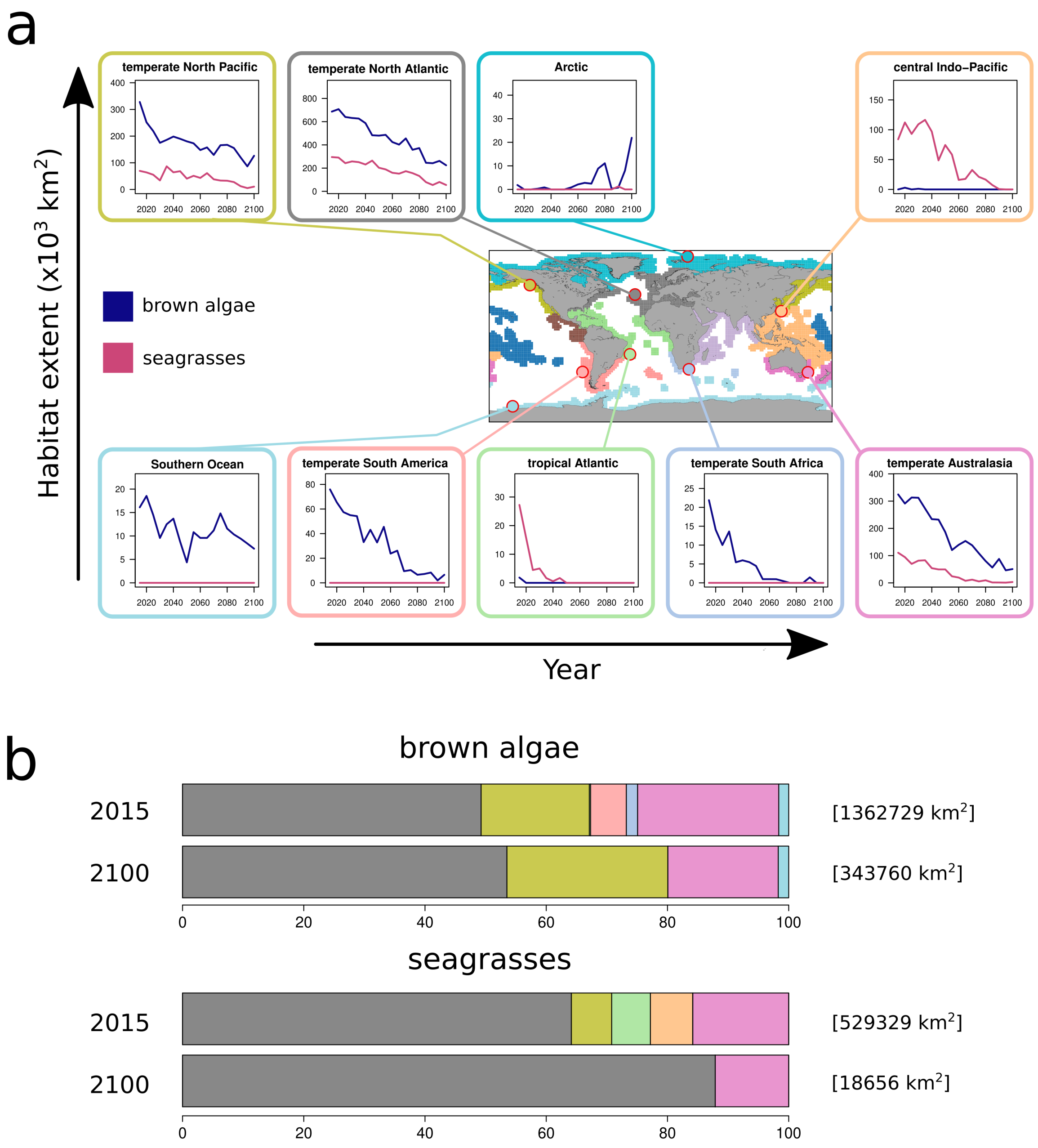

Supplementary Fig. 9 Variation in the extent of suitable macrophyte habitat ($p > 0.9$) across marine regions (emissions scenario SSP3-7.0). We calculated global macrophyte habitat suitability using a machine-learning approach (see Methods) for the period 2015–2100, and applied a threshold of $p = 0.9$ to select only highly suitable macrophyte habitat (only marine regions where highly suitable macrophyte habitat is present shown). **a** Variation in macrophyte habitat extent (km^2) for brown macroalgae (purple) and seagrasses (pink) within each marine region, aggregated every 5 years. **b** Comparison of the percentage of global suitable macrophyte habitat in each marine region between 2015 and 2100 for brown macroalgae (upper bar plots) and seagrasses (lower bar plots).

Colours refer to marine regions as shown in **a**. Square brackets show total global suitable habitat extension. The map in **a** was generated using the package *Basemap* in Python 3.

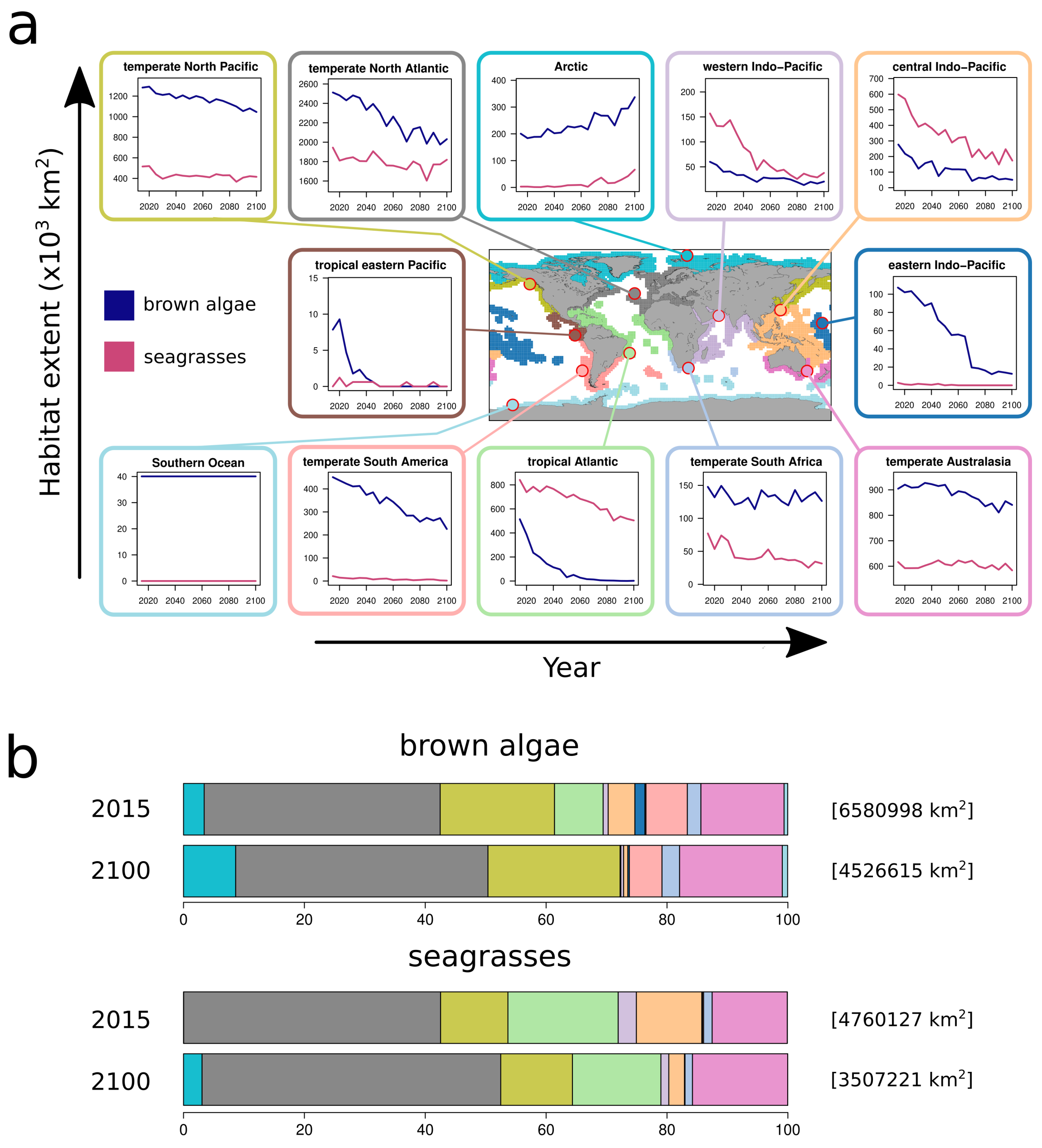

Supplementary Fig. 10 Variation in the extent of suitable macrophyte habitat ($p > 0.6$) across marine regions (emissions scenario SSP2-4.5). We calculated global macrophyte habitat suitability using a machine-learning approach (see Methods) for the period 2015–2100, and applied a threshold of $p = 0.6$ to ensure that all regions included at least one suitable grid cell. **a** Variation in macrophyte habitat extent (km^2) for brown macroalgae (purple) and seagrasses (pink) within each marine region, aggregated every 5 years. **b** Comparison of the percentage of global suitable macrophyte habitat in each marine region between 2015 and 2100 for brown macroalgae (upper bar plots) and seagrasses (lower bar plots). Colours refer to marine regions as shown in **a**. Square

brackets show total global suitable habitat extension. The map in **a** was generated using the package *Basemap* in Python 3.

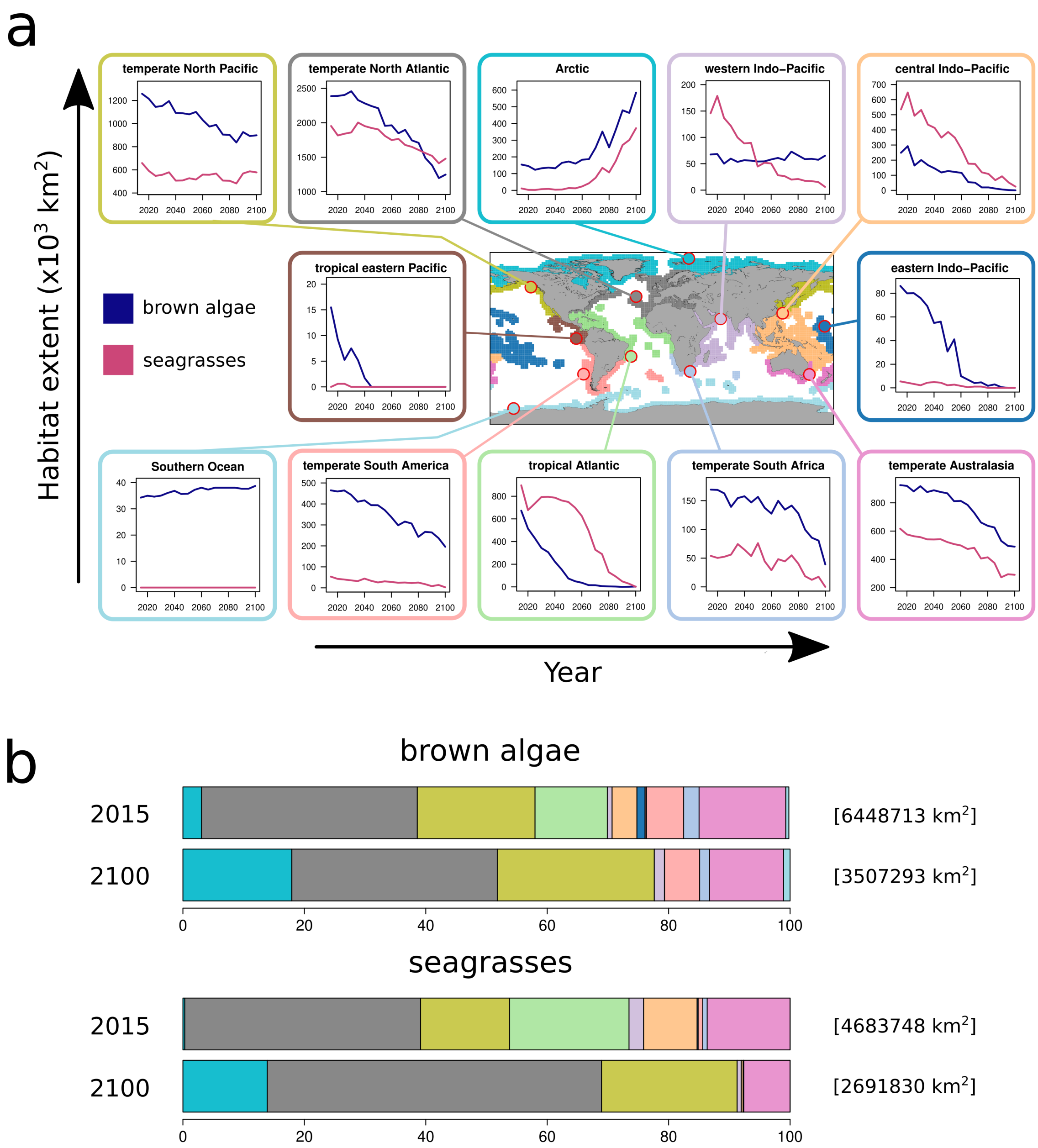

Supplementary Fig. 11 Variation in the extent of suitable macrophyte habitat (p > 0.6) across marine regions (emissions scenario SSP5-8.5). We calculated global macrophyte habitat suitability using a machine-learning approach (see Methods) for the period 2015–2100, and applied a threshold of p = 0.6 to ensure that all regions included at least one suitable grid cell. **a** Variation in macrophyte habitat extent (km^2) for brown macroalgae (purple) and seagrasses (pink) within each marine region, aggregated every 5 years. **b** Comparison of the percentage of global suitable macrophyte habitat in each marine region between 2015 and 2100 for brown macroalgae (upper bar plots) and seagrasses (lower bar plots). Colours refer to marine regions as shown in **a**. Square

brackets show total global suitable habitat extension. The map in **a** was generated using the package *Basemap* in Python 3.

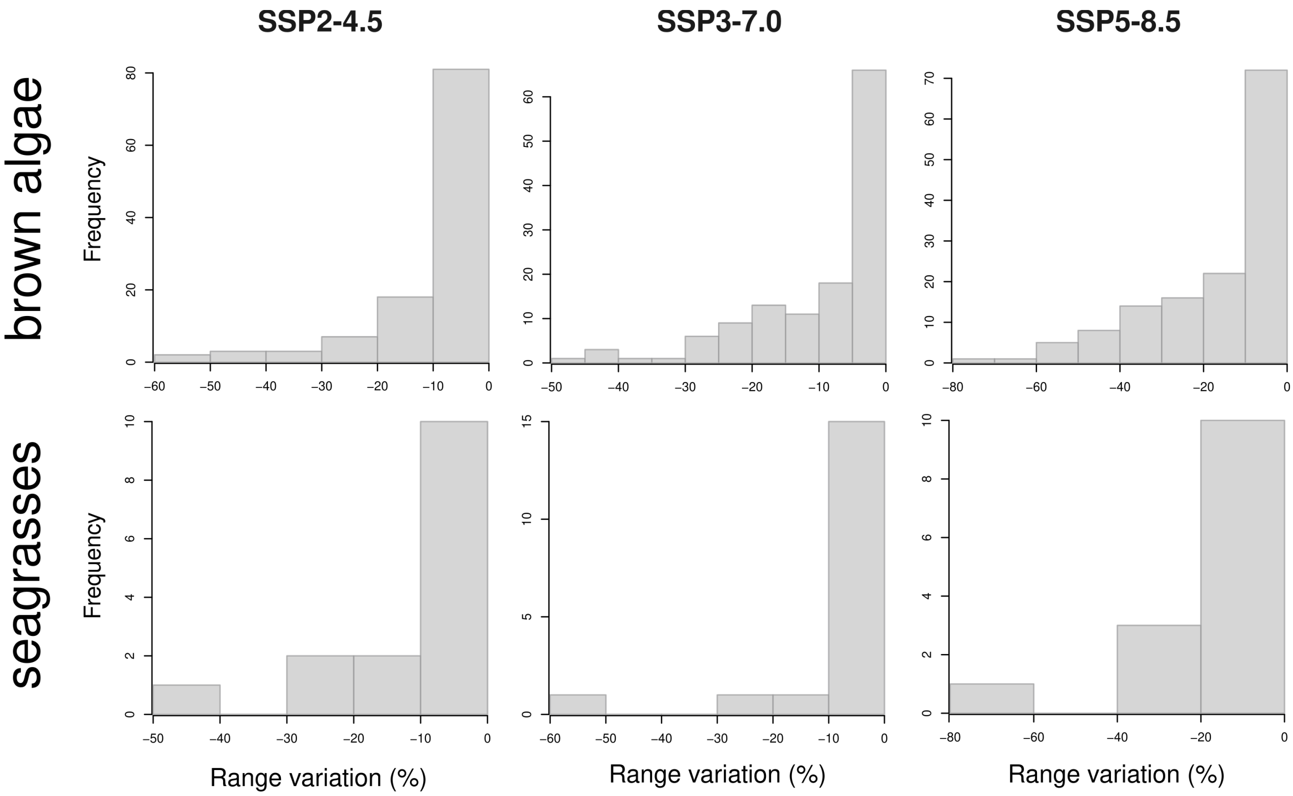

Supplementary Fig. 12 Distribution of macrophyte area-of-occupancy erosion by 2100. Most species of brown macroalgae and seagrasses are expected to lose a small percentage of their present area of occupancy ("range" in the figure for simplicity). The upper panels show brown macroalgae (185 species), the lower panels show seagrasses (22 species). Columns correspond to different emissions scenarios.

Supplementary Table 1 List of the ten most impacted marine macrophyte species. Macrophyte species that are expected to face the highest losses in area-of-occupancy (hereafter "range" for simplicity) extension by 2100, according to our model projections. For each species, the present (2015), future (2100) range extension, relative end-of-century range change, and environmental predictors selected in the models (in decreasing order of importance) are reported under different emissions scenarios (SSP2-4.5, SSP3-7.0, and SSP5-8.5).

Emission scenario	Species	Kingdom	Range 2015 (km^2)	Range 2100 (km^2)	Relative range change (%)	Environmental predictors
SSP5-8.5	*Laminaria yezoensis*	Chromista	58931.87	12673.05	-78.5	sea surface temperature, air temperature, salinity, radiation, primary productivity, sea-ice cover
SSP5-8.5	*Zostera muelleri subsp. capricorni*	Plantae	184140.67	41398.41	-77.52	sea surface temperature, radiation, air temperature, salinity, primary productivity, depth, sea-ice cover
SSP5-8.5	*Laminaria digitata*	Chromista	312683.93	122082.24	-60.96	sea surface temperature, radiation, air temperature, salinity, primary productivity, sea-ice cover, depth
SSP2-4.5	*Laminaria ochroleuca*	Chromista	144853.37	58208.09	-59.82	radiation, air temperature, salinity, sea surface temperature, primary productivity, depth
SSP5-8.5	*Laminaria bullata*	Chromista	2033.17	926.61	-54.43	salinity, sea surface temperature, air temperature, radiation, primary productivity, sea-ice cover
SSP5-8.5	*Tilopteris mertensii*	Chromista	344403.81	158371.43	-54.02	radiation, air temperature, sea surface temperature, salinity, primary productivity, sea-ice cover
SSP3-7.0	*Zostera muelleri subsp. capricorni*	Plantae	171031.01	79368.93	-53.59	sea surface temperature, radiation, air temperature, salinity, primary productivity, depth, sea-ice cover
SSP5-8.5	*Saccharina nigripes*	Chromista	184132.75	85731.58	-53.44	sea surface temperature, air temperature, radiation, salinity, sea-ice cover, primary productivity
SSP2-4.5	*Bifurcaria bifurcata*	Chromista	283524.97	133647.13	-52.86	radiation, air temperature, salinity, sea surface temperature, primary productivity, sea-ice cover, depth
SSP5-8.5	*Bifurcaria bifurcata*	Chromista	284447.86	137763.54	-51.57	radiation, air temperature, salinity, sea surface temperature, primary productivity, depth

References

1. Gaston, K. J. & Fuller, R. A. The sizes of species' geographic ranges. *J. Appl. Ecol.* **46**, 1–9 (2009).
2. Törnqvist, L., Vartia, P. & Vartia, Y. O. How should relative changes be measured? *Am. Stat.* **39**, 43–46 (1985).
