# Supplementary material for: Projected loss of brown macroalgae and seagrasses with global environmental change
Source: Nat Commun. 2024 Jun 24;15:5344. doi: 10.1038/s41467-024-48273-6 (PMC11196678; doi:10.1038/s41467-024-48273-6)
Supplement: Supplementary file 3 — Description of Additional Supplementary Files [file 41467_2024_48273_MOESM3_ESM.pdf]

Description of Additional Supplementary Files

Supplementary Data 1: List of brown macroalgal species modelled in the study. For each species, the present-day (2015), future (2100) area-of-occupancy (hereafter "range" for simplicity) extension (km^2), relative end-of-century range change (%), and environmental variables selected in the models are reported under three emissions scenarios (SSP2-4.5, SSP3-7.0, SSP5-8.5). Environmental variables are reported in decreasing order of importance (as mean decrease impurity).

Supplementary Data 2: List of seagrass species modelled in the study. For each species, the present (2015), future (2100) area-of-occupancy (hereafter "range" for simplicity) extension (km^2), relative end-of-century range change (%), and environmental variables selected in the models are reported under three emissions scenarios (SSP2-4.5, SSP3-7.0, SSP5-8.5). Environmental variables reported in decreasing order of importance (as mean decrease impurity).
