# Supplementary material for: Projected loss of brown macroalgae and seagrasses with global environmental change
Source: Nat Commun. 2024 Jun 24;15:5344. doi: 10.1038/s41467-024-48273-6 (PMC11196678; doi:10.1038/s41467-024-48273-6)
Supplement: Supplementary file 6 — Reporting Summary [file 41467_2024_48273_MOESM6_ESM.pdf]

nature portfolio

Corresponding author(s): Federica Manca

Last updated by author(s): Mar 29, 2024

Reporting Summary

Nature Portfolio wishes to improve the reproducibility of the work that we publish. This form provides structure for consistency and transparency in reporting. For further information on Nature Portfolio policies, see our Editorial Policies and the Editorial Policy Checklist.

Statistics

For all statistical analyses, confirm that the following items are present in the figure legend, table legend, main text, or Methods section.

n/a	Confirmed	
[ ]	[x]	The exact sample size (n) for each experimental group/condition, given as a discrete number and unit of measurement
[x]	[ ]	A statement on whether measurements were taken from distinct samples or whether the same sample was measured repeatedly
[x]	[ ]	The statistical test(s) used AND whether they are one- or two-sided<br>*Only common tests should be described solely by name; describe more complex techniques in the Methods section.*
[ ]	[x]	A description of all covariates tested
[x]	[ ]	A description of any assumptions or corrections, such as tests of normality and adjustment for multiple comparisons
[ ]	[x]	A full description of the statistical parameters including central tendency (e.g. means) or other basic estimates (e.g. regression coefficient) AND variation (e.g. standard deviation) or associated estimates of uncertainty (e.g. confidence intervals)
[x]	[ ]	For null hypothesis testing, the test statistic (e.g. F, t, r) with confidence intervals, effect sizes, degrees of freedom and P value noted<br>*Give P values as exact values whenever suitable.*
[x]	[ ]	For Bayesian analysis, information on the choice of priors and Markov chain Monte Carlo settings
[x]	[ ]	For hierarchical and complex designs, identification of the appropriate level for tests and full reporting of outcomes
[x]	[ ]	Estimates of effect sizes (e.g. Cohen's d, Pearson's r), indicating how they were calculated

Our web collection on statistics for biologists contains articles on many of the points above.

Software and code

Policy information about availability of computer code

Data collection	We downloaded the data manually from the sources listed in the "Data" section below.
Data analysis	We analyzed data using custom code written in Python 3 and R version 4.2.1. The code permitting to fully replicate the analyses is available at https://github.com/mancfede/macrophytes_global. Spatial data manipulation and analysis was performed using the Geospatial Data Abstraction Library (GDAL), Rasterio, Scipy, and Shapely in Python 3. For the random forest classifier we used the package Scikit-learn in Python 3.

For manuscripts utilizing custom algorithms or software that are central to the research but not yet described in published literature, software must be made available to editors and reviewers. We strongly encourage code deposition in a community repository (e.g. GitHub). See the Nature Portfolio guidelines for submitting code & software for further information.

Data

Policy information about availability of data

All manuscripts must include a data availability statement. This statement should provide the following information, where applicable:

- Accession codes, unique identifiers, or web links for publicly available datasets
- A description of any restrictions on data availability
- For clinical datasets or third party data, please ensure that the statement adheres to our policy

The data used in this study were retrieved from the following sources: (1) macrophyte occurrence records: https://figshare.com/articles/dataset/A_fine-tuned_global_distribution_dataset_of_marine_forests/7854767; (2) environmental predictors (mean monthly sea surface temperature, surface air temperature,

surface salinity, surface primary productivity, and sea-ice cover): Coupled Model Intercomparison Project Phase 6 (cera-www.dkrz.de/WDCC/ui/cerasearch/cmip6?input=CMIP6.ScenarioMIP.DKRZ.MPI-ESM1-2-HR.ssp845 and wdc-climate.de/ui/cmip6?input=CMIP6.ScenarioMIP.CNRM-CERFACS.CNRM-CM6-1-HR.ssp370); (3) monthly incoming shortwave radiation: Eumestat Climate Modelling (CM) Satellite Application Facility (https://wui.cmsaf.eu); (4) irradiance at sea bottom: Bio Oracle (https://bio-oracle.org); (5) depth: GEBCO (https://www.gebco.net/). All the data used and generated in this study have been deposited in Zenodo with the identifier 10.5281/zenodo.10371401 [https://doi.org/10.5281/zenodo.10371401].

Research involving human participants, their data, or biological material

Policy information about studies with human participants or human data. See also policy information about sex, gender (identity/presentation), and sexual orientation and race, ethnicity and racism.

Reporting on sex and gender	N/A
Reporting on race, ethnicity, or other socially relevant groupings	N/A
Population characteristics	N/A
Recruitment	N/A
Ethics oversight	N/A

Note that full information on the approval of the study protocol must also be provided in the manuscript.

Field-specific reporting

Please select the one below that is the best fit for your research. If you are not sure, read the appropriate sections before making your selection.

☐ Life sciences ☐ Behavioural & social sciences ☒ Ecological, evolutionary & environmental sciences

For a reference copy of the document with all sections, see nature.com/documents/nr-reporting-summary-flat.pdf

Ecological, evolutionary & environmental sciences study design

All studies must disclose on these points even when the disclosure is negative.

Study description	We modelled the future global distribution of brown macroalgae and seagrasses. We combined occurrence data of brown macroalgae and seagrasses and a set of environmental and climatic predictors at the global scale to map both 'generic' brown macroalgal and seagrass habitats (i.e., habitat suitable to host any brown macroalgal or seagrass species, regardless of species identity), and the distribution of 207 individual macrophyte species. We derived maps for both the present and the future (from 2015 to 2100, at a yearly temporal resolution) under three greenhouse gas-emissions scenarios (Shared Socio-economic Pathways 73 SSP2-4.5, SSP3-7.0, and SSP5-8.5). We explored future trajectories of change in macrophyte habitat extension and species diversity, as well as in individual species' ranges. We estimate that by 2100, local macrophyte diversity will decline by 3–4% on average, with 17 to 22% of localities losing at least 10% of their macrophyte species globally. The current range of macrophytes will be eroded by 5–6%, and highly suitable macrophyte habitat will be substantially reduced globally (78–96%). Global macrophyte habitat will shift among marine regions, with a high potential for expansion in polar regions.
Research sample	We used global datasets of the occurrence of brown macroalgae and seagrasses and of different environmental and climatic predictors (sea surface temperature, surface air temperature, surface salinity, surface primary productivity, surface incoming shortwave radiation, irradiance at bottom, sea-ice cover and depth).
Sampling strategy	We did not predetermine sampling size.
Data collection	We collected all the data from the following sources: (1) macrophyte occurrence records: https://figshare.com/articles/dataset/A_fine-tuned_global_distribution_dataset_of_marine_forests/7854767; (2) environmental predictors (mean monthly sea surface temperature, surface air temperature, surface salinity, surface primary productivity, and sea-ice cover): Coupled Model Intercomparison Project Phase 6 (cera-www.dkrz.de/WDCC/ui/cerasearch/cmip6?input=CMIP6.ScenarioMIP.DKRZ.MPI-ESM1-2-HR.ssp845 and wdc-climate.de/ui/cmip6?input=CMIP6.ScenarioMIP.CNRM-CERFACS.CNRM-CM6-1-HR.ssp370); (3) monthly incoming shortwave radiation: Eumestat Climate Modelling (CM) Satellite Application Facility (https://wui.cmsaf.eu); (4) irradiance at sea bottom: Bio Oracle (https://bio-oracle.org); (5) depth: GEBCO (https://www.gebco.net/).
Timing and spatial scale	Data were collected from online sources between March and August 2022. All data were obtained at the global scale.
Data exclusions	From the full list of occurrence records of brown macroalgae and seagrasses, we excluded all species having less than 10 occurrence records, resulting in a final list of n=185 species of brown macrolagae and n=22 seagrass species. This allowed focusing on species for which sufficient occurrence data exist to make reliable predictions.
Reproducibility	All the data used in the study are available in Zenodo with the identifier 10.5281/zenodo.10371401. The code permitting to fully replicate the analyses is available at https://doi.org/10.5281/zenodo.1090766487.
Randomization	Our study design did not require randomization.

Blinding | We used third party data, hence blinding was not relevant for our study.

Did the study involve field work? ☐ Yes ☒ No

Reporting for specific materials, systems and methods

We require information from authors about some types of materials, experimental systems and methods used in many studies. Here, indicate whether each material, system or method listed is relevant to your study. If you are not sure if a list item applies to your research, read the appropriate section before selecting a response.

Materials & experimental systems

n/a	Involved in the study
☒	☐ Antibodies
☒	☐ Eukaryotic cell lines
☒	☐ Palaeontology and archaeology
☒	☐ Animals and other organisms
☒	☐ Clinical data
☒	☐ Dual use research of concern
☒	☐ Plants

Methods

n/a	Involved in the study
☒	☐ ChIP-seq
☒	☐ Flow cytometry
☒	☐ MRI-based neuroimaging

Plants

Seed stocks	N/A
Novel plant genotypes	N/A
Authentication	N/A
